# Characterization of the complete chloroplast genome of *Salix maizhokunggarensis* (Salicaceae)

**DOI:** 10.1080/23802359.2020.1721374

**Published:** 2020-02-03

**Authors:** Rui Ma, Mijuan Zhou, Yujie Wang, Guoyu Zhu, Junyi Li, Qiuhong Feng, Ning Miao

**Affiliations:** a Key Laboratory of Bio-Resource and Eco-Environment of Ministry of Education/College of Life Sciences, Sichuan University, Chengdu, Sichuan, P. R. China;; b Forestry and Grassland Bureau of Hongyuan County, Hongyuan, Sichuan, P. R. China;; c State Key Laboratory of Hydraulics and Mountain River Engineering/College of Water Resource and Hydropower, Sichuan University, Chengdu, Sichuan, P. R. China;; d Sichuan Academy of Forestry/Ecological Restoration and Conservation on Forest and Wetland Key Laboratory of Sichuan Province, Chengdu, Sichuan, P. R. China

**Keywords:** *Salix maizhokunggarensis*, Salicaceae, chloroplast genome, phylogenetic tree

## Abstract

The complete chloroplast genome sequence of *Salix maizhokunggarensis*, a native shrub willow species in the south of China, has been characterized using Illumina pair-end sequencing. The plastome is 155,093 bp in length, with one large single copy region of 83,956 bp, one small single copy region of 16,221 bp, and two inverted repeat (IR) regions of 27,458 bp. It contains 116 genes, including 79 protein-coding genes, 8 ribosomal RNA, and 36 transfer RNA. Phylogenetic tree shows that this species is a sister species to *S. suchowensis*. The plastome of *Salix* can provide significant insight for elucidating the phylogenetic relationship of taxa within Salicaceae.

*Salix maizhokunggarensis* N. Chao belongs to genus *Salix* in the family of Salicaceae is a native shrub willow species in the south of China (Fang et al. 1999; Ohashi, 2001). The fresh fruits can be used for medicine and cosmetic. Because of the complexity of their inter- and intraspecific morphological variations, species delimitation in genus *Salix* is quite difficult (Mirski 2014). A well-resolved phylogeny based on sufficient molecular markers is essential to understanding the relationship between species, and the efficient utilization and improvement of these wild *Salix* species as crop. In this study, we reported the complete chloroplast genome of *S. maizhokunggarensis*, which is a widely distributed species in the temperate areas of China, as source for future study on taxonomy of *Salix* genus.

Fresh leaves of *S. maizhokunggarensis* were collected in Milashan Mountains (Mozhugongka County, Tibet, China; Coordinates: 92°20′E, 29°54′N) and dried with silica gel. The voucher specimen was stored in Sichuan University Herbarium with the accstion number of 20170904001. Total genomic DNA was extracted with a modified CTAB method (Doyle and Doyle 1987). First, we obtained 10 million high-quality pair-end reads for *S. maizhokunggarensis*, and after removing the adapters, the remained reads were used to assemble the complete chloroplast genome by NOVOPlasty (Dierckxsens et al. 2017). The complete chloroplasts genome sequence of *S. babylonica* was used as a reference. Plann v1.1 (Huang and Cronk 2015) and Geneious v11.0.3 (Kearse et al. 2012) were used to annotate the chloroplasts genome and correct the annotation.

The total plastome length of *S. maizhokunggarensis* (MN952983) is 155,093 bp, exhibits a typical quadripartite structural organization, consisting of a large single copy (LSC) region of 83,956 bp, two inverted repeat (IR) regions of 27,458 bp and a small single copy (SSC) region of 16,221 bp. The cp genome contains 116 complete genes, including 79 protein-coding genes (79 PCGs), 8 ribosomal RNA genes (4 rRNAs), and 36 tRNA genes (38 tRNAs). Most genes occur in a single copy, while 12 genes occur in double, including 7 tRNAs (trnA-UGC, trnI-CAU, trnI-GAU, trnL-CAA, trnN-GUU, trnR-ACG, and trnV-GAC), and 5 PCGs (rps19, rpl2, rpl23, ndhB, ycf2). The overall GC content of cp DNA is 36.7%, the corresponding values of the LSC, SSC, and IR regions are 34.5, 31.0, and 41.9%.

In order to further clarify the phylogenetic position of *S. maizhokunggarensis*, plastome of 12 representative Salicaceae species were obtained from NCBI to construct the plastome phylogeny, with *Populus trichocarpa* as an outgroup. All the sequences were aligned using MAFFT v.7.313 (Katoh and Standley 2013) and maximum likelihood phylogenetic analyses were conducted using RAxML v.8.2.11 (Stamatakis 2014). The phylogenetic tree shows that *Salix* clade were identified two subclades: *S. paraplesia*, *S. babylonica,* and *S. chaenomeloides* are belonged to one clustered, while *S. maizhokunggarensis, S. suchowensis, S. rehderiana,* and *S. taoensis* clustered together with *S. purpurea* in this subclade (Figure 1).

**Figure 1.**
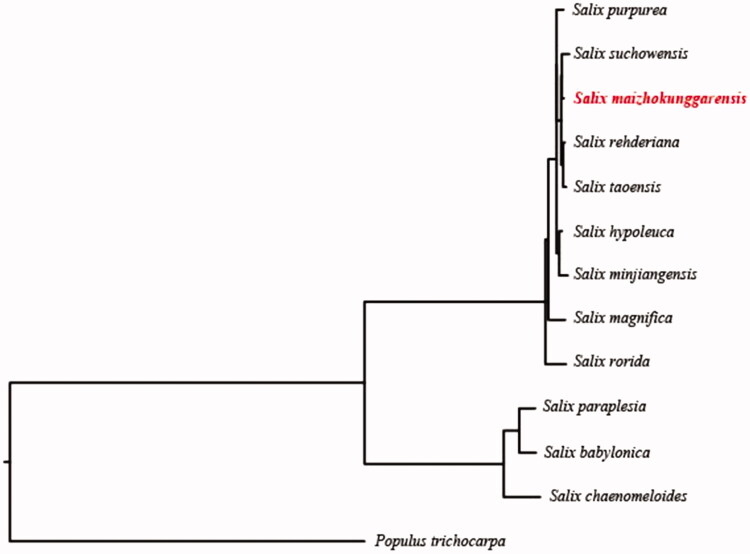
Phylogenetic relationships of Salicaceae species using whole chloroplast genome. GenBank accession numbers: *Salix purpurea* (NC_026722), *Salix suchowensis* (NC_026462), *Salix maizhokunggarensis* (MN952983)*, Salix rehderiana* (MG262367), *Salix taoensis* (MG262369), *Salix hypoleuca* (MG262363), *Salix minjiangensis* (MG262365), *Salix magnifica* (MG262364), *Salix rorida* (MG262368), *Salix paraplesia* (MG262366), *Salix babylonica* (MG262361), *Salix chaenomeloides* (MG262362), and *Populus trichocarpa* (NC_009143).
